# Association between cigarette smoking, *APC *mutations and the risk of developing sporadic colorectal adenomas and carcinomas

**DOI:** 10.1186/1471-2407-6-71

**Published:** 2006-03-17

**Authors:** Mona Sæbø, Camilla F Skjelbred, Rebecca Breistein, Inger Marie B Lothe, Per Chr Hagen, Gunter Bock, Inger-Lise Hansteen, Elin H Kure

**Affiliations:** 1 Telemark University College, Faculty of Arts and Sciences, Hallvard Eikas plass, 3800 Bø i Telemark, Norway; 2 Department of Laboratory Medicine, Telemark Hospital, 3710 Skien, Norway; 3 Department of Surgery, Telemark Hospital, 3710 Skien, Norway; 4 Department of Pathology, Ulleval University Hospital, Kirkeveien 166, 0407 Oslo, Norway

## Abstract

**Background:**

The association between colorectal cancer (CRC) and smoking has not been consistent. Incomplete smoking history and association to a specific subset of CRC tumors have been proposed as explanations. The adenomatous polyposis coli (*APC*) gene has been reported to have a "gatekeeper" function in the colonic mucosa.

**Methods:**

To evaluate the hypothesis that cigarette smoking is associated with adenoma and carcinoma development and further to investigate whether this association is due to mutations in the *APC *gene, we used a study population consisting of 133 cases (45 adenomas and 88 carcinomas) and 334 controls. All tumors were sequenced in the mutation cluster region (MCR) of the *APC *gene. Cases and controls were drawn from a homogeneous cohort of Norwegian origin.

**Results:**

The mutational spectra of the *APC *gene revealed no difference in frequencies of mutations in cases based on ever and never smoking status. An overall case-control association was detected for adenomas and "ever smoking" OR = 1.73 (95% CI 0.83–3.58). For CRC cases several smoking parameters for dose and duration were used. We detected an association for all smoking parameters and "duration of smoking > 30 years", yielded a statistically significant OR = 2.86 (1.06–7.7). When cases were divided based on *APC *truncation mutation status, an association was detected in adenomas without *APC *mutation in relation to "ever smoking", with an OR = 3.97 (1.26–12.51). For CRC cases without *APC *mutation "duration of smoking > 30 years", yielded a statistically significant OR = 4.06 (1.20–13.7). The smoking parameter "starting smoking ≥ 40 years ago" was only associated with CRC cases with *APC *mutations, OR = 2.0 (0.34–11.95). A case-case comparison revealed similar findings for this parameter, OR = 2.24 (0.73–6.86).

**Conclusion:**

Our data suggest an association between smoking and adenoma and CRC development. This association was strongest for cases without *APC *truncation mutation. This may implicate other factors in development of these tumors. The association detected between smoking and CRC cases with *APC *mutation was in relationship to the smoking parameter "starting smoking ≥ 40 years ago", a time period long enough to proceed CRC initiation.

## Background

Development of sporadic colorectal adenomas and carcinomas has been associated with several lifestyle factors, including cigarette smoking [1,2]. Cigarette smoke contains a large number of carcinogens, which may bind to DNA and form adducts, potentially causing irreversible genetic damage in the normal colorectal mucosa [3-5]. The large bowel is exposed to these compounds through the circulatory system [1], and it has been proposed that the first hit in colorectal cancer (CRC) development is most likely caused by blood-borne carcinogens in the colonic crypt [6]. This makes carcinogens from tobacco smoke a potential source. A number of case control studies have shown a strong correlation between cigarette smoking and the risk of developing adenomas, but the evidence linking cigarette smoking and CRC has been inconsistent [1]. Cigarette smoking as the initial cause of CRC may require a smoking history as long as 40 years [1,7]. In studies conducted prior to 1970 only a limited number of smokers would have exceeded four decades of smoking. This has been proposed as an explanatory factor for the inconsistent results in linking cigarette smoking to CRC development [1].

Another explanation for this discrepancy is that cigarette smoking is only involved in tumors with specific genetic mutations. So far, the genetic targets of carcinogens from cigarette smoking in CRC development have not been fully elucidated. It is possible that several genes in different molecular pathways are affected. CRC development may take at least two different pathways based on genomic instability. The majority of sporadic cases display chromosomal instability (CIN) while microsatellite instability (MSI) with mutations in mismatch repair genes is displayed in about 15% [8]. CRC tumors displaying MSI have been positively associated with cigarette smoking in four studies while one study detected no such association [9-13]. The CIN pathway involves mutations in several genes including adenomatous polyposis coli (*APC*), *k-ras *and *TP53*. As a target gene for initiation, *APC *fits the profile with its "gatekeeper" function in the colonic mucosa [14-16]. *APC *is a tumor suppressor gene in the Wnt signaling pathway, and it regulates cell proliferation mainly through its association with β-catenin [17]. Mutations in the *APC *gene have been detected in the majority of adenoma and CRC cases [16,18-23]. The so called "mutation cluster region" (MCR), codons 1286 – 1513, represents <10% of the *APC *gene, but previous studies have shown that as much as 60–70% of the somatic mutations occur in this region [20,21,24,25]. A truncation mutation in the MCR region of the *APC *gene will result in a protein that lacks most of its 20-amino acids β-catenin binding and down regulation sites.

Only two case-control studies have to our knowledge investigated the association between cigarette smoking and *APC *truncation mutations in CRC development [10,11]. They reported no association when focusing on "ever smoking", "starting smoking > 35 years ago", "high dose or long duration", but interestingly they detected an inverse association for "starting smoking < 35 years ago" [11], and "first starting smoking after age 25" [10]. A smoking parameter of "years since starting smoking", which precedes the initiating point for CRC development, might therefore be important when evaluating *APC *mutation and CRC risk.

Most studies have focused on truncation mutations. Even though the biological significance of missense and silent mutations are uncertain, the mutational spectra of the *APC *gene may provide a more complete picture of impact and type of carcinogenic exposure.

In this Norwegian case-control study, the KAM cohort, we used direct sequencing of the *APC *MCR to evaluate the mutational spectra of the gene in relation to smoking history in both colorectal adenomas and carcinomas. We also investigated the overall adenoma and CRC risk in association with several smoking parameters. The same investigation was further conducted after dividing the case groups based on *APC *truncation mutation status with a particular focus on CRC and years since starting smoking.

## Methods

The KAM cohort (Kolorektal cancer, Arv og Miljø) is based on the screening group of the Norwegian Colorectal Cancer Prevention study (The NORCCAP study) in Telemark [26], and a series of colorectal cancer cases from the same region (Telemark) operated on at Telemark Hospital in Skien. The KAM biobank has previously been described [27]. The KAM cohort is based on an ethnic homogeneous group of Norwegian origin. The ID number for the NORCCAP study at Clinicaltrials.gov is – I NCT00119912 [28]. Those invited to participate in the NORCCAP study were 20,780 men and women, age 50–64 years old, drawn by randomization from the population registry. The overall attendance rate was 65%. The 48 adenoma cases with severe degree of dysplasia and a control group of 334 individuals were drawn from the NORCCAP study. All the controls in the KAM cohort that completed the questionnaire were included. The controls were screen negative participants (negative flexible sigmoidoscopy). For CRC cases, all patients diagnosed with colorectal cancer who were mentally competent to complete the questionnaire were asked to participate in the KAM study. The questionnaire contained information on a family history of cancer and the included CRC cases had no known personal history of cancer. In this study 94 CRC cases were drawn from the KAM cohort. The tumor histology of the carcinomas and adenomas was examined independently by two specialist histopathologists in order to determine the tumor stage. The Regional Ethics Committee and the Data Inspectorate have approved the KAM study.

Sequencing was performed on DNA isolated from formalin-fixed, paraffin-embedded colon tumor tissue, collected prior to chemo- or radiotherapy treatment (> 60% tumor cells) using a GenoPrep™ DNA Isolation from Tissue kit (GenoVision). The analysis covers codons 1260 – 1585, including all the codons in the extended MCR, residues 1281 – 1556 [29]. The reference sequence for human *APC *is Genbank Accession Number M74088. The MCR region was divided into five overlapping fragments and amplified separately in two consecutive PCRs. The first fragment is 297 bp, codons 1260 – 1358. The PCR primers are: Fragment 1.1: (1246–1358) forward – AAGTGGTCAGCCTCAAAAGG, reverse – CGCTCCTGAAGAAAATTCAAC, and fragment 1.2: (1260–1358) forward – CAGACTTATTGTGTAGAAG, reverse CGCTCCTGAAGAAAATTCAAC. The PCR reaction mixture (total volume 50 μl) contained 50 ng DNA, 0.4 μM of each primers, 0.2 mM dNTP's, 1× PCR buffer, 2–3 mM MgCl_2 _and 0.5 U *Taq *DNA polymerase. The reaction conditions for the first PCR: Fragment 1.1: 5 cycles of 30 s at 94°C, 45 s at 56°C, 1 min 70°C, followed by 25 cycles of 30 s at 94°C, 45 s at 54°C, 1 min 70°C followed by 5 min at 70°C. The primers and reaction conditions for the second PCR and remaining four fragments, each 220 bp long, and primers and reaction conditions have been described elsewhere [18]. All products from the five different fragments were purified with Min Elute PCR Purification Kit (Qiagen) before sequencing. The sequencing was performed on ABI 310 automatic sequencer using Big Dye Terminator v 1.1 Cycle Sequencing kit (Applied) by standard protocol and primers from the second PCR. In 10 of the CRC cases and 8 of the adenoma cases one or more fragments of the sequenced region of the *APC *gene could not be amplified. Some of these cases had mutations in the amplified fragments and these mutations were included in evaluating the mutational spectra of the gene. For case-control comparison all cases with detected truncation mutation were used. Cases that showed no mutations and or failed in amplification were left out of the data analysis.

In order to validate the sensitivity of our sequence analysis of paraffin-embedded tissues, we compared analysis performed on the same samples using DNA from both blood and paraffin-embedded tissue on a specific region of the *APC *gene. The G4497A silent polymorphism is located in the MCR of *APC*. We used the amplified products from fragment 4.2 and the restriction enzyme *BtgI *to cut the wild type allele in order to identify samples that were homozygot for the wild type and the polymorphism, respectively. The cutting conditions were 1× NE-buffer, 0.3 mM *BtgI *with a total volume of 11 μl and 4 μl of PCR product. The samples were incubated over night at 37°C. The detection limit of the mutational analysis was determined by mixing the homozygous PCR product for the polymorphic allele with increasing concentrations of the corresponding wild type allele, and sequencing these mixtures on the ABI 310 as described for the samples.

All of the participants were requested to fill in a self-administered questionnaire on dietary and personal history, including smoking habits and family history of cancer. Smoking habits were assessed as current smoking status, number of cigarettes smoked per day, total number of years smoked, age of starting smoking, and if applicable the age at which the participant stopped smoking [30-32].

Differences in characteristics between groups were assessed using the χ^2^test for categorical variables and the Mann-Whitney test for continuous variables; p values < 0.05 were considered significant. We examined by logistic regression the association between smoking and development of colorectal adenomas and carcinomas measured separately as odds ratio (OR) with 95% confidence interval (CI). The controls used as a reference group in this study of adenomas and carcinomas are matched to the cases by region (Telemark). An overall case-control comparison was conducted as well as separately comparing cases with or without *APC *truncation mutation (APC ^+ ^and APC ^-^, respectively), to the polyp-free controls in relationship to smoking history. Case-case comparison was conducted to evaluate heterogeneity in risk factors. The risk factors evaluated were: cigarette smoking status (never, ever), number of cigarettes smoked per day (≤15; > 15), duration of smoking in years (≤ 30; > 30), and years since first starting smoking (≥ 40 years ago). MiniTab Statistical Software, Release 13.1 Xtra (Minitab Inc.) was used for statistical calculations. The data are age and sex adjusted.

## Results

### Study population

Selected characteristics of the controls, and all cases with satisfactory amplification and mutation analysis of the *APC *are given in Table 1.

### Mutation analysis

The sensitivity of the mutational analysis, sequenced on the ABI 310-genetic analyzer, was independent of whether the DNA originated from blood or paraffin-embedded tissue. We were able to detect the polymorphic allele at 20% polymorphic PCR product in a background of wild-type PCR product.

*APC *mutations were detected in 55% of the adenomas (25/45) and in 51% of the CRC tumors (45/88). *APC *truncation mutations were detected in 40% of the adenomas (18/43) and in 32% of the CRC tumors (27/84), respectively. Only 10 of the tumors (5 adenomas and 5 CRC, respectively) had more than one mutation detected. None of the tumors had more than two mutations detected. A polymorphism ACG → ACA (G4497A) which does not alter the amino acid (threonine) was observed in 71% (32/45) of the adenoma cases and 71% (62/88) of the CRC cases, respectively.

The number of cases with mutations according to ever and never smoking status are 44/88 (50%) and 22/39 (56%), respectively. Some of these cases may have more than one mutation. Type and distribution of all detected mutations are displayed in Table 2. All mutations are displayed together since we detected no statistical significant difference between the adenoma and CRC case groups in relation to frequency and mutational spectra (data not shown).

Characteristics of the adenoma and CRC cases with or without truncation mutations only are given in Table 3.

### Case-control

Ever smoking was not significantly associated with increased overall adenoma risk (see Table 4). When dividing the adenoma case group based on *APC *truncational status "ever smoked" was significanly associated with APC ^- ^adenomas only, OR = 3.97.57 (CI 1.26–12.51). Due to small sample size of the adenoma case group, statistics for the other smoking parameters were not calculated.

"Duration of smoking > 30 years" was statistically significantly associated with increased overall CRC risk, OR 2.86 (1.06–7.7). For CRC cases divided based on *APC *truncation mutation status all the smoking characteristics, exept for "starting smoking ≥ 40 years ago", yielded a higher odds ratio for APC ^- ^tumors compared to APC ^+ ^tumors (see Table 5).

## Discussion

In this study direct sequencing was used to identify adenomas and CRC tumors with or without any *APC *mutations in the MCR region of the gene, and the results were compared to history of smoking. The overall adenoma and CRC risk as well as cases divided based on *APC *truncation mutation status, were assessed according to several smoking parameters.

We detected fewer *APC *silent/missens mutations than a resent Dutch study did [33], but the frequencies of truncated mutations detected for CRC and adenomas are comparable to published studies on *APC *truncation mutations (30–45%) [11,16,18,21,23,33]. The location and type of mutations detected (data not shown) are also consistent with those reported in the *APC *database [34]. The KAM study is to the best of our knowledge the first study to evaluate the association between colorectal adenomas, *APC *mutations and smoking history, and also to evaluate the complete mutational spectra of *APC *in CRC cases in relation to smoking history.

We detected no significant difference between the adenoma and CRC case groups, or between ever and never smokers in relation to frequency and type of mutation. A Japanese study reported similar *APC *truncation mutations frequencies [35]. With the exception of one A → T transversion all the truncated point mutations detected were G:C → T:A transversions and C → T transitions. The silent/missense mutations displayed a more heterogenous mutational spectra.

For overall case-control comparison a statistically significant association was detected for CRC cases and "duration of smoking > 30 years", OR = 2.86 (CI 1.06–7.7). The fact that the CRC cases and controls have not been matched by age may affect the result of the analysis, but the result is comparable to other studies published in recent years [1]. The selection of controls is vital, and our controls have all been screened in the colon and found to be polyp free at flexible sigmoidoscopy. It has been estimated that the risk of erroneously classifying individuals with proximal advanced neoplasia as neoplasia-free is less than 3% at flexible sigmoidoscopy [36]. No significant association was observed for adenomas and smoking history. This may be due to low sample size.

When dividing adenoma cases based on *APC *truncation mutation status a statistically significant association was detected for "ever smoked" and APC^- ^adenomas only. This implies the involvment of other genes in association with cigarette smoking and adenoma development. Both the MSI pathway and other CIN genes such as *k-ras *have previously been associated with cigarette smoking and CRC development [9-13]. The results may also indicate the importance of the smoking parameter when evaluating *APC*. If *APC *truncation mutations are involved in the initiating process, this association may be lost or diminished when using a parameter without time frame of exposure. For adenomatous polyps both latency period for adenoma development, and which ones will develop into cancerous tumors is difficult to predict. It is interesting that when comparing the adenoma and CRC case groups to different smoking parameters, mean years since starting smoking is the only parameter that is statistically significantly different between the two case groups, *P *< 10^-4^. Although, our adenoma case group is small, all have been diagnosed with severe degree of dysplasia. The advanced adenoma, defined by high-grade dysplasia, size > 1 cm or villous features, has a greater potential of developing into a cancerous tumor than small adenomas with mild or moderate dysplasia [37].

For CRC cases divided based on *APC *truncation mutational status all smoking parameters, except "starting smoking ≥ 40 years ago", were associated with APC^- ^cases. For "duration of smoking > 30 years" this association was statistically significant, OR = 4.06 (1.20–13.7). For APC ^+ ^cases no significant association was detected, but "duration of smoking > 30 years" and "starting smoking ≥ 40 years ago" showed an association. This may indicate that cigarette smoking is associated with tumors without *APC *truncation mutation, as reported in two previous published studies by Diergaarde *et al *[11] and Lûchtenborg *et al *[10]. These findings are comparable to the obtained results for adenomas. As previously discussed, this may imply the involvment of other genes in association with cigarette smoking and colorectal carcinogenesis. Sporadic CRC is a complex disease and several environmental factors in combination with genetic make up may have an impact on CRC development. The chromosomal instability (CIN) pathway involves several genes including *APC*, *k-ras *and *TP53 *[14-16]. It has been reported that smoking may be associated with *k-ras *transversion mutations and play a role in *TP53 *negative tumors in CRC [11]. The other major pathway for CRC development involves microsatellite instability (MSI) [8]. Slattery et *al *reported that MSI positive cases are more likely to smoke more than 20 sigarettes a day [9] and CRC tumors displaying MSI have been positively associated with cigarette smoking.

However, smoking parameters like amount and duration of smoking may not be the correct measures in validating initiating causes for CRC development. Diergaarde *et al *also reported a statistically significant inverse association between CRC cases with *APC *truncation mutation and "starting smoking ≤ 35 years ago" (OR 0.02, CI 0.1–0.8) [11], and Lûchtenborg *et al *reported an inverse association between "age first smoked ≥ 25 years" and CRC cases with *APC *truncation mutation (OR 0.54, CI 0.26–1.10) [10]. These results indicate that a sufficiently long time since first exposed to cigarette smoke is important in order to evaluate the association of smoking, *APC *mutation and CRC development. For cigarette smoking to be the initiating factor a latency period of approximately 40 years has been proposed [7]. In this study the case-case (APC^+ ^versus APC^-^) comparison of CRC cases showed no association or an inverse association for all smoking parameters, except "starting smoking ≥ 40 years ago" with an OR = 2.24 (CI 0.73–6.86). This result is in concordance with the theory of *APC *truncation mutations in the initiating phases of CRC development. In addition, "starting smoking ≥ 40 years ago" was the only smoking parameter that was significantly different for APC ^+ ^and APC^- ^CRC cases, *P *= 0.010. This parameter (starting smoking ≥ 40 years ago) highlights the importance of the particular point in time when starting smoking. There was no difference between the two CRC subgroups when comparing dose and duration.

## Conclusion

Our results indicate that there is an association between cigarette smoking and adenoma and CRC development. For cases divided based on *APC *truncation mutation status this association was strongest for cases without mutation. This indicates that there are other factors that may play a major role in development of these tumors. The exception was for a smoking history with a time span of 40 years or more since first starting smoking. This smoking parameter was only associated with cases with *APC *mutations, though this association was not statistically significant. This may suggest that smoking can contribute to CRC development through mutations in the *APC *gene if smoking starts prior to CRC initiation. A larger study would be required to clarify this issue.

## Competing interests

The author(s) declare that they have no competing interests.

## Authors' contributions

MS extracted samples, performed the sequencing analysis on the *APC *gene and prepared the first draft of the paper. She did the data analysis. CFS contributed with technical advice on the sequencing of the *APC *gene and design of primers. She also contributed to the manuscript and with advice in the data analysis. RB performed in the sequencing of the adenomas and sensitivity analysis. IMBL did the pathology including the selection of sections for DNA extraction. PCH contributed with statistical advice. GB collected tumor tissues. ILH participated in collection and quality control of the questionnaires. EHK brought the idea and organized the study. She contributed with advice on the data analysis and was responsible for the revisions of the paper. All authors discussed the results, contributed to interpretation of the results and the final manuscript.

## Pre-publication history

The pre-publication history for this paper can be accessed here:



## Figures and Tables

**Table 1:** Distribution of selected characteristics by case-control status.

		Cases	
		
Characteristics	Controls n = 334	Adenoma cases n = 45	CRC cases n = 88
Men^a^	129 (39%)	29 (65%)	53 (60%)
Women^a^	205 (61%)	16 (35%)	35 (40%)
Mean age at cohort entry^b^	54.3 ± 3.4	57.5 ± 3.6	68.1 ± 9.7
Mean body mass index. kg/m^b^	26.05 ± 3.7	27.2 ± 5.7	25.0 ± 3.8
Smoking parameters*			
Ever smoked^c^	53% (177/334)	73% (32/44)	67% (56/83)
Mean no. of cigarettes per day^c^	10.64 ± 7.3	13.1 ± 6.5	12.7 ± 8.7
Mean no. of years of smoking^c^	22.0 ± 11.3	33.6 ± 10.3	33.3 ± 15.0
Mean years since starting smoking^d^	34.3 ± 6.8	39.2 ± 6.2	47.0 ± 8.7

*Missing values for smoking parameters gave rise to diminished numbers of cases.

^a^There are significant differences in no. of males and females among the control group and the adenoma and CRC case groups, *P *= 0.001 and < 10^-4 ^respectively, but no difference between the two case groups *P *= 0.64.

^b^There are significant differences in age between the control group and both of the case groups, and between the adenoma and CRC case groups, *P *< 10^-4^.

^c^There are significantly more ever smokers in the adenoma and CRC case groups compared to the controls, *P *= 0.013 and 0.017, respectively, but no significant difference between the case groups *P *= 0.54. There is no significant difference between the control group and the adenoma and CRC case groups in number of cigarettes smoked per day, *P *= 0.07 and 0.15, respectively. There are significant differences between the control group and both of the case groups for duration of smoking *P *< 10^-4^.

^d^There are significant differences in years since starting smoking between the control group and both of the case groups, and also between the adenoma and CRC case groups, *P *< 10^-4^.

**Table 2:** *APC *mutational spectra detected in all adenoma/CRC cases, and cases divided according to smoking status.

Type of mutation^a^	All cases n = 133 Total number of mutations	Ever smokers^*b ^n = 88		Never smokers^*b ^n = 39	
		
		Silent/missense mutation	Truncation mutation	Silent/missense mutation	Truncation mutation
Transitions	31(39%)	13 (72%)	7 (25%)	7 (50%)	2 (13%)
C → T	17	2	7	4	2
G → A	9	6	-	3	-
A → G	4	4	-	-	-
T → C	1	1	-	-	-
Transversion	21(26%)	5 (28%)	5 (18%)	7 (50%)	2 (13%)
A → C	-	-	-	-	-
T → G	2	1	-	1	-
A → T	1	-	1	-	-
T → A	4	2	-	2	-
C → A	3	1	2	-	-
G → T	6	1	2	1	2
C → G	-	-	-		-
G → C	3	-	-	3	-
Frameshift	28 (35%)	-	16 (57%)	-	11 (74%)
Total	80	18	28	14	15

*Missing values for ever and never smoking gave rise to diminished numbers of cases and mutations.

^a ^The frequency is calculated by dividing the number of transitions, transversions and framshift mutations separately to the total number of either silent/missens or truncated mutations for ever and never smokers.

^b^There is no significant difference between ever smokers and never smokers in relation to the mutational spectra (transitions, transversions and frameshift) for missens/silent or truncated mutations *P *= 0.094 and 0.558, respectively.

**Table 3:** Distribution of selected smoking characteristics for adenoma and carcinoma cases divided by *APC *truncation-mutation status.

	Cases*			
	Adenomas		Carcinomas	
	
Characteristics^a^	APC^+ ^(n = 18)	APC^- ^(n = 25)	APC^+ ^(n = 27)	APC^- ^(n = 57)
Ever smoked	59% (10/17)	84% (22/26)	68% (17/25)	68% (37/54)
Mean no. cigarettes per day	10,5 ± 5,3	14.5 ± 7,4	11,3 ± 10,0	13,5 ± 8,4
Mean years smoking	39,2 ± 4,6	30,9 ± 11,5	32,4 ± 15,2	34,2 ± 15,1
Mean years since started smoking	40,3 ± 4,8	38,9 ± 6,9	48,9 ± 6,2	46,3 ± 9,8
Starting smoking ≥ 40 years ago	60% (6/10)	45% (10/22)	100%(17/17)	69% (25/36)

*Uncertain *APC *truncation mutation status for one or more of the sequenced fragments gave rise to diminished numbers of CRC and adenoma cases, compared to the study population in Table 1.

^a^None of the smoking parameters were significantly different within the adenoma group. Within the CRC group only "starting smoking ≥ 40 years ago" yielded a significant difference, *P *= 0.010.

**Table 4:** Adenomas, smoking history and *APC *truncation mutation status.

Smoking parameters^a^	All adenomas^b ^versus controls	APC^+ ^versus controls	APC^- ^versus controls	APC^+^versus APC^-^
Never smoked§				
Case/control	13/157	*6/157	*4/157	*6/4*
Ever smoked				
Case/control	34/177	*11/177	*22/177	11/22
OR (95% CI)	1.73 (0.83–3.58)	0.96 (0.33–2.85)	**3.97 (1.26–12.51)**	0.24 (0.05–1.11)

*Missing values for "ever smoked" gave rise to diminished numbers of adeoma cases.

§ Never smoked serves as the reference category.

^a^All the OR's are adjusted for age and sex.

^b^For overall case-control comparison of adenoma status and smoking all the selected adenoma cases from the study population that had completed the questionnaire for smoking parameters were used 47/48.

**Table 5:** Colorectal cancer, smoking history and *APC *truncation mutation status.

Smoking Parameters ^a^	CRC cases versus controls ^b^	APC^+ ^versus controls	APC^- ^versus controls	APC^+ ^versus APC^-^
Never smoked§				
Case/control	30/157	*8/157	*17/157	8/17
Ever smoked				
Case/control	58/177	*17/177	*37/177	17/37
OR (95% CI)	1.46 (0.63–3.36)	0.54 (0.09–3.24)	2.31 (0.81–6.60)	0.82 (0.26–2.60)
≤15 cigarettes smoked per day				
Case/control	42/137	*13/137	*26/137	13/26
OR (95% CI)	1.32 (0.55–3.16)	0.08 (0.34–3.21)	2.17 (0.74–6.39)	1.00 (0.32–3.15)
> 15 cigarettes smoked per day				
Case/control	15/38	*4/38	*10/38	4/10
OR (95% CI)	2.21 (0.67–7.28)	0.74 (0.07–7.94)	3.04 (0.71–13.0)	0.70 (0.14–3.50)
Smoking ≤ 30 years				
Case/control	*25/27	*9/123	*16/123	9/16
OR (95% CI)	1.74 (0.65–4.63)	0.07 (0.00–1.11)	1.59 (0.49–5.08)	0.99 (0.26–3.80)
Smoking > 30 years				
Case/control	30/52	*8/52	*20/52	8/20
OR (95% CI)	**2.86 (1.06–7.7)**	2.45 (0.36–16.7)	**4.06 (1.20–13.7)**	0.77 (0.22–2.68)
Never smoked or starting smoking < 40 years ago§§				
Case/control	42/306	*8/306	*28/306	8/28
Starting smoking ≥ 40 years ago				
Case/control	45/27	*17/27	*25/27	17/25
OR (95% CI)	1.10 (0.40–3.01)	2.0 (0.34–11.95)	0.93 (0.29–3.01)	2.24 (0.73–6.86)

*Missing values for smoking charactristics gave rise to diminished numbers of CRC cases.

§ Never smoked was used as reference category for "ever smoked", "dosage ≤ 15, > 15" and "duration ≤ 30, > 30".

§§ The combination of never smoked and starting smoking <40 years ago, was used as reference category for "starting smoking ≥ 40 years ago".

^a^All the OR's are adjusted for age and sex.

^b^For overall case-control comparison of CRC status and smoking, all the selected cases from the study population that had completed the questionnaire for smoking parameters were used.
